# Mucosa-associated lymphoid tissue lymphoma translocation protein 1 exaggerates multiple organ injury, inflammation, and immune cell imbalance by activating the NF-κB pathway in sepsis

**DOI:** 10.3389/fmicb.2023.1117285

**Published:** 2023-03-07

**Authors:** Yane Wang, Zhimin Liu, Mengli Zhang, Bo Yu, Fen Ai

**Affiliations:** ^1^Department of Emergency, The Central Hospital of Wuhan, Tongji Medical College, Huazhong University of Science and Technology, Wuhan, China; ^2^Department of Thyroid and Breast Surgery, The Central Hospital of Wuhan, Tongji Medical College, Huazhong University of Science and Technology, Wuhan, China

**Keywords:** mucosa-associated lymphoid tissue lymphoma translocation protein 1, sepsis, multiple organ injury, immune cell imbalance, nuclear factor-κB pathway

## Abstract

**Objective:**

Mucosa-associated lymphoid tissue lymphoma translocation protein 1 (MALT1) modulates the inflammatory immune response and organ dysfunction, which are closely implicated in sepsis pathogenesis and progression. This study aimed to explore the role of MALT1 in sepsis-induced organ injury, immune cell dysregulation, and inflammatory storms.

**Methods:**

Septic mice were constructed by intraperitoneal injection of lipopolysaccharide, followed by overexpression or knockdown of MALT1 by tail vein injection of the corresponding lentivirus. Mouse naïve CD4^+^ T cells and bone marrow-derived macrophages were treated with MALT1 overexpression/knockdown lentivirus plus lipopolysaccharide.

**Results:**

In the lungs, livers, and kidneys of septic mice, MALT1 overexpression exaggerated their injuries, as shown by hematoxylin and eosin staining (all *p* < 0.05), elevated cell apoptosis, as reflected by the TUNEL assay and cleaved caspase-3 expression (*p* < 0.05 in the lungs and kidneys), and promoted macrophage infiltration, as illustrated by CD68 immunofluorescence (*p* < 0.05 in the lungs and kidneys). Meanwhile, in the blood, MALT1 overexpression reduced T-helper (Th)1/Th2 cells, increased Th17/regulatory T-cell ratios (both *p* < 0.05), promoted systematic inflammation, as revealed by tumor necrosis factor-α, interleukin-6, interleukin-1β, and C-reactive protein (all *p* < 0.05), elevated oxidative stress, as shown by nitric oxide (*p* < 0.05), superoxide dismutase, and malondialdehyde (*p* < 0.05), and enhanced liver and kidney dysfunction, as revealed by an automatic animal biochemistry analyzer (all *p* < 0.05 except for aspartate aminotransferase). However, MALT1 knockdown exerted the opposite effect as MALT1 overexpression. *Ex vivo* experiments revealed that MALT1 overexpression promoted the polarization of M1 macrophages and naïve CD4^+^ T cells toward Th2 and Th17 cells (all *p* < 0.05), while MALT1 knockdown attenuated these effects (all *p* < 0.05). Mechanistically, MALT1 positively regulated the nuclear factor-κB (NF-κB) pathway both *in vivo* and *ex vivo* (*p* < 0.05).

**Conclusion:**

Mucosa-associated lymphoid tissue lymphoma translocation protein 1 amplifies multiple organ injury, inflammation, oxidative stress, and imbalance of macrophages and CD4^+^ T cells by activating the NF-κB pathway in sepsis.

## 1. Introduction

Sepsis is characterized by a dysregulated inflammatory immune response toward infection, which commonly causes multiple organ injury, such as lung, liver, kidney, etc., and even death (Singer et al., 2016; Cecconi et al., 2018). It has been reported that sepsis affects almost 49 million individuals and induces 11 million deaths worldwide annually (Rudd et al., 2020); moreover, it is also estimated that the mortality rate of sepsis in the intensive care unit (ICU) reaches as high as 41.9% (Fleischmann-Struzek et al., 2020). In addition, sepsis also generates a tremendous burden on the health care system, both financially and in terms of humanity (Rudd et al., 2018; van den Berg et al., 2022). Currently, the management of sepsis mainly includes antimicrobial therapy, fluid therapy, vasoactive therapy, mechanical ventilation, etc (Alhazzani et al., 2020; Gavelli et al., 2021; Burgunder et al., 2022). However, these therapies only ameliorate disease symptoms but cannot effectively relieve the outburst of inflammatory storms and immune dysregulation in sepsis. Therefore, it is vital to explore potential targets involved in the pathogenesis and progression of sepsis, which may identify latent treatments for sepsis.

The dysregulation of immune cells, such as macrophages and T helper (Th) cells, is critically involved in sepsis (Chen et al., 2021; Wang et al., 2022). For instance, the M1 polarization of macrophages releases a great amount of proinflammatory cytokines, including tumor necrosis factor-α (TNF-α), interleukin-1β, and IL-6. During sepsis, it also mediates multiple organ injury in sepsis (Jin et al., 2022; Li et al., 2022; Zhang et al., 2022). In terms of Th cells, studies have implied that Th2 and Th17 cells are aberrant, and they are associated with unfavorable prognosis in septic patients (Gupta et al., 2016; Zhao et al., 2021). Preclinical findings also suggest that the imbalance of Th1/Th2 and Th17/regulatory T (Treg) also facilitates inflammation in sepsis (Rendon and Choudhry, 2012; Brady et al., 2020).

Mucosa-associated lymphoid tissue lymphoma translocation protein 1 (MALT1) is a paracaspase that is closely involved in the inflammatory immune response and organ dysfunction. For instance, MALT1 promotes the differentiation of CD4^+^ T cells into Th17 cells by activating the nuclear factor-κB (NF-κB) pathway (Wang et al., 2022). Another study proposed that knockdown of MALT1 induces a lower level of Th17 cells but not Th1 cells and suppresses inflammation in colitis mice (Nakamura et al., 2019). Moreover, inhibition of MALT1 reduces injuries to the lung, liver, kidney, and brain (McAllister-Lucas et al., 2007; Fusco et al., 2020; Marko et al., 2020; Zhang et al., 2021). In terms of the involvement of MALT1 in sepsis, a recent clinical study revealed that MALT1 is elevated in septic patients, and its high expression is positively correlated with Th1 cells, multiple organ injury, and mortality risk in septic patients (Wang et al., 2022). However, the regulation of MALT1 in the pathogenesis and progression of sepsis is still unclear.

In the current study, a septic mouse model was constructed by injection of lipopolysaccharide (LPS) followed by modification of MALT1 in a septic mouse model. Meanwhile, MALT1 was modified in primary macrophages and naïve CD4^+^ T cells from mice, followed by LPS treatment. The current study aimed to explore the effect of MALT1 on multiple organ injuries, macrophage infiltration and polarization, Th1/Th2 and Th17/Treg imbalance, and its downstream pathway in sepsis.

## 2. Methods

### 2.1. Animals

C57BL/6 mice (6 weeks old, 20 ± 2 g) were provided by Shanghai SLAC Laboratory Animal Co., Ltd (Shanghai, China) and housed in a pathogen-free environment. All animal studies were approved by the animal ethics committee of the Central Hospital of Wuhan and performed following the guidelines of our institute.

### 2.2. Lipopolysaccharide-induced septic mouse model

A total of 12 mice were divided into the Sham group and Model group [n = 6 in each group, sample size was decided according to previous studies (Shi et al., 2021; Li et al., 2022)]. The mice in the Model group received an intraperitoneal injection of lipopolysaccharide (LPS, from *Escherichia coli* O55:B5, L6529, Sigma, United States) at 30 mg/kg (which was in accordance with a previous study (Li et al., 2022) and verified in a preliminary study) to construct a septic mouse model, and the mice in the Sham group were given an intraperitoneal injection of the same volume of phosphate buffered solution (PBS).

Afterward, MALT1 overexpression (LV-MALT1), MALT1 knockdown (LV-shMALT1), or negative control lentivirus (LV-NC) were purchased from GenePharma (Shanghai, China) to evaluate the influence of MALT1 on septic mice. Briefly, 24 mice were divided into four groups: the Model, LV-NC, LV-MALT1, and LV-shMALT1 groups (*n* = 6 per group). The LV-NC, LV-MALT1, and LV-shMALT1 groups received an injection of the corresponding lentivirus through the tail vein (5 × 10^8^ TU/mL in 100 μL PBS), and the mice in the Model group were given an injection of the same volume of PBS. After being injected for 2 weeks (Ding et al., 2021), LPS-induced septic mice were constructed as mentioned above.

All mice were sacrificed by cervical dislocation after anesthesia at 24 h after LPS injection. Peripheral blood, lung, liver, and kidney samples were collected for further detection. Meanwhile, the spleen and bone marrow samples of the Sham group were collected for naïve CD4^+^ T-cell and bone marrow-derived macrophage (BMDM) culture, respectively.

A survival experiment was performed to establish a successful septic phenotype. Briefly, 12 mice were divided into the Sham group (*n* = 6) and Model group (*n* = 6), and injection of LPS in the Model group was performed as mentioned above. The living condition of mice was observed every 4 h. All mice were grouped using a computer-based random order generator in this study. No inclusion or exclusion criteria were set. No strategy was used to avoid potential confounding factors. No humane endpoint was set.

### 2.3. Cell culture, transfection, and lipopolysaccharide treatment

Naïve CD4^+^ T cells were isolated from spleen samples of mice with a naïve CD4 T-cell isolation reagent (Invitrogen, United States) and maintained in T-cell culture medium (Takara, Japan). BMDMs were isolated as described previously (Tang et al., 2020) and maintained in DMEM (Servicebio, China) plus 10% FBS (Avantor, China), 1% penicillin–streptomycin (Servicebio, China) and 20 ng/mL M-CSF (Sigma, United States). Naïve CD4^+^ T cells and BMDMs were transfected with LV-NC, LV-MALT1, and LV-shMALT1 according to the manufacturer’s instructions. After 72 h of transfection, 1 μg/mL LPS was added to treat naïve CD4^+^ T cells and BMDMs. The cells without transfection and LPS treatment served as the control group. After another 24 h of treatment, reverse-transcription quantitative PCR (RT–qPCR) and western blotting were carried out, and the cell supernatant of BMDMs was collected for ELISA.

### 2.4. Th2 and Th17 cell polarization

For polarization of Th2 and Th17 cells, treated naïve CD4^+^ T cells were plated into 24-well plates (5 × 10^4^ cells/well) and stimulated with different polarizing conditions (Wu et al., 2018). Briefly, anti–IFN-γ (20 μg/mL; Abcam, United Kingdom) and IL-4 (20 ng/mL; Sigma, United States) were added for Th2 cell polarization. For Th17 cell polarization, anti-IL-4 (10 μg/mL; Abcam, United Kingdom), anti–IFN-γ (10 μg/mL), IL-6 (30 ng/mL; Sigma, United States), IL-1β (10 ng/mL; Santa Cruz, China), TGF-β (2 ng/mL; Santa Cruz, China), and IL-23 (20 ng/mL; Santa Cruz, China) were used. The stimulated cells were harvested for flow cytometry after 3 days of stimulation.

### 2.5. Hematoxylin and eosin and TUNEL staining

The lung, liver, and kidney samples of mice were fixed using fixative (Solarbio, China), embedded using HistoCore Arcadia (Leica, German) and cut into 4 μm-thick sections using RM2016 (Leica, German). HE and TUNEL staining was carried out using HE staining solution (Servicebio, China) and a TUNEL Detection Kit (Beyotime, China), respectively. The injury score was analyzed according to HE staining as described in a previous study (Li et al., 2022) by investigators who were unaware of treatment. Alveolar septum thickening, renal tubular epithelial cell swelling, inflammatory cell infiltration, hepatocyte swelling, focal necrosis, and diffuse coagulation were included in the manifestations of multiple organ injury. The injury areas were scored as follows: 0 for normal, 1 for <25% damage, 2 for 25–50% damage, 3 for 50–75% damage, 4 for 75–90% damage, and 5 for >90% damage.

### 2.6. Immunohistochemistry and immunofluorescence staining

For IHC staining, the sections were incubated with anti-cleaved-caspase3 (1:200; Affinity, China) or anti-p-NF-κB p65 (1:100; Affinity, China) antibodies and then incubated with the goat anti-rabbit secondary antibody (1:200; Affinity, China). The sections were then stained with DAB (Servicebio, China) and hematoxylin (Servicebio, China). For IF staining, the sections were incubated with anti-CD68 (1:50; Affinity, China) antibody, and the sections were incubated with a goat anti-rabbit IgG (H + L) cy3-conjugated secondary antibody (1:200; Affinity, China). Then, DAPI (Invitrogen, United States) staining was performed.

### 2.7. Detection of immunological and biochemical indexes

Serum was collected from the peripheral blood of mice by centrifugation (3,000 rpm, 15 min). The expression of TNF-α, IL-6, IL-1β, IL-8, IL-10, and c-reactive protein (CRP) in serum and the TNF-α, IL-6, and IL-1β levels in the cell supernatant of BMDMs were assessed using ELISA kits (Sangon, China) according to the kits’ instructions. Nitric oxide (NO), superoxide dismutase (SOD), and malondialdehyde (MDA) were detected using an NO detection kit (Solarbio, China), SOD detection kit (Jiancheng, China), and MDA detection kit (Jiancheng, China) in accordance with the kits’ instructions. The levels of serum creatinine (SCR), blood urea nitrogen (BUN), alanine aminotransferase (ALT), aspartate aminotransferase (AST), and lactate dehydrogenase (LDH) were detected using an automatic animal biochemistry analyzer (SYSMEX, Japan).

### 2.8. Flow cytometry

PBMCs were isolated from the peripheral blood of mice with PBMC isolation reagent (NovoBio, China). The proportions of Th1, Th2, Th17, and Treg cells were detected with Mouse Th1/Th2/Th17/Treg detection kits (Multi Sciences, China). Briefly, PBMCs were treated with PMA/Ionomycin reagent (Multi Sciences, China) and BFA/Monensin reagent (Multi Sciences, China) for 6 h and stained with the corresponding antibodies. Cells were then detected by a FACS flow cytometer (BD, United States), and data processing was accomplished by FlowJo software (V7.6; FlowJo, United States). The mentioned method was also used to detect Th2 and Th17 cell proportions in naïve CD4^+^ T cells after polarization.

### 2.9. RT–qPCR

RNA was mined from mice (peripheral blood, lung, liver, and kidney samples) and cells (BMDMs and naïve CD4^+^ T cells) using TRIzol (Invitrogen, United States). RT–qPCR was performed using a cDNA synthesis kit (Takara, Japan) and PCR kit (Takara, Japan). The relative MALT1 expression was analyzed using the 2^−ΔΔCt^ method. The primers were as follows (5′-3′): MALT1 (forward, GCCTGGACCTGGAACAGTGT; reverse, AGAAGCGGAAGAACCTCAGTGT) and GAPDH (forward, AACAGCAACTCCCACTCTTC; reverse, CCTGTTGCTGTAGCCGTATT).

### 2.10. Western blot

Total protein from BMDMs, naïve CD4^+^ cells, peripheral blood, lung, liver, and kidney was extracted with RIPA lysis reagent (Servicebio, China). The protein was separated by SDS–PAGE on 4–20% precast gels (Willget, China) and transferred to nitrocellulose filter membranes. The membranes were incubated with blocking buffer (1 h, 37°C), primary antibodies (1 h, 37°C), and goat anti-rabbit or goat anti-mouse secondary antibodies (1:5000; Affinity, China; 1 h, 37°C). The protein signals were detected with an ECL reagent (Servicebio, China). The primary antibodies used were as follows: anti-MALT1 (1:2000; Abcam, United Kingdom), anti-iNOS (1:200; Santa Cruz, China), anti-p-NF-κB p65 (1:500; Affinity, China), anti-NF-κB p65 (1:200; Santa Cruz, China), and anti-GAPDH (1:5000; Affinity, China).

### 2.11. Statistical analysis

Data analysis was implemented with GraphPad Prism (Ver7.0; GraphPad, United States). Student’s t test or Mann–Whitney U test were used to analyze significance between two groups. One-way ANOVA plus Tukey’s *post hoc* test was adopted for comparisons among groups. The normality was analyzed by Shapiro–Wilk test. Kaplan–Meier survival plots and log-rank tests were used to compare survival between groups. A *p* value <0.05 was set as statistically significant.

## 3. Results

### 3.1. Mucosa-associated lymphoid tissue lymphoma translocation protein 1 was elevated in the septic mouse model

After injection with LPS, tissue samples of the lung, liver, and kidney were acquired for HE, TUNEL, and cleaved-caspase3 detection (Supplementary Figures S1A–C). Then, quantitative analyses revealed that compared with Sham group, the injury scores (all *p* < 0.001; Supplementary Figures S1D–F), TUNEL-positive rates (all *p* < 0.01; Supplementary Figures S1G–I), and cleaved-caspase3 IHC scores (all *p* < 0.05; Supplementary Figures S1J–L) of the lung, liver, and kidney were all increased in Model group. Meanwhile, the detection of macrophage infiltration by IF (Supplementary Figure S2A) revealed that CD68-positive cells in the lung, liver, and kidney were higher in the Model group vs. Sham group (all *p* < 0.001; Supplementary Figures S2B–D). In addition, the serum levels of inflammatory cytokines (including TNF-α, IL-6, IL-1β, IL-8, and IL-10) and CRP were elevated (Supplementary Figures S2E–J); the oxidative stress markers NO and MDA were reduced, but the antioxidative stress enzyme SOD was increased (Supplementary Figures S2K–M); and the liver and kidney dysfunction markers LDH, SCR, BUN, ALT, and AST (Supplementary Figures S2N–R) were all enhanced in the Model group vs. Sham group (all *p* < 0.05). In addition, the survival rate was lower in the Model group vs. Sham group (*p* < 0.01; Supplementary Figure S3). These data suggested the success of LPS-induced septic mouse model construction.

**Figure 1:**
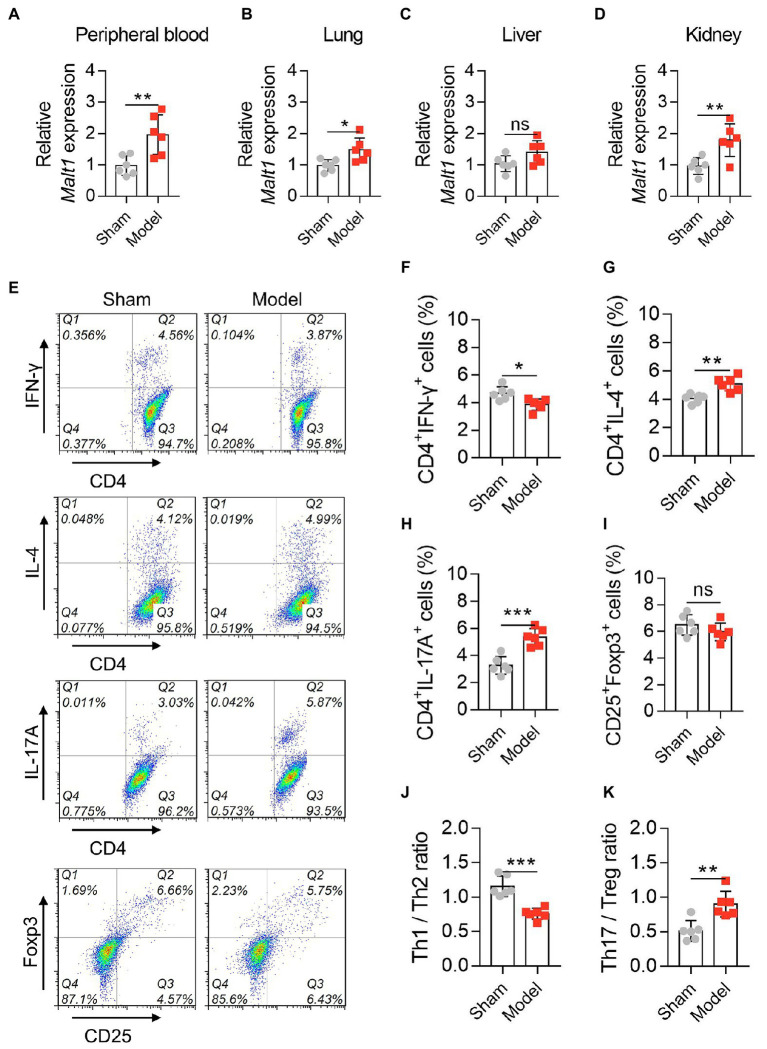
MALT1 and CD4^+^ T cells in LPS-induced septic mouse model. Comparison of *Malt1* expression in peripheral blood **(A)**, lung **(B)**, liver **(C)**, and kidney **(D)** between the Model group and Sham group. Detection of CD4^+^ T cells by flow cytometry **(E)**. Comparison of Th1 **(F)**, Th2 **(G)**, Th17 **(H)**, and Treg **(I)** cells, Th1/Th2 ratio **(J)**, and Th17/Treg ratio **(K)** between the Model group and Sham group. *n* = 6 in each group. Student’s *t* test was applied. Ns, not significant; *p < 0.05; **p < 0.01; ***p < 0.001.

Next, *Malt1* was detected by RT–qPCR, which revealed that *Malt1* in the peripheral blood (*p* < 0.01), lung (*p* < 0.05), and kidney (*p* < 0.01) was elevated in the Model group vs. Sham group, while it was not different in the liver (*p* > 0.05) between them (Figures 1A–D). Moreover, data from flow cytometry (Figure 1E) showed that the Model group had a lower level of Th1 cells (*p* < 0.05; Figure 1F) but higher levels of Th2 cells (*p* < 0.01; Figure 1G) and Th17 cells (*p* < 0.001; Figure 1H) vs. Sham group; however, Treg cells remained unchanged between groups (*p* > 0.05; Figure 1I). Furthermore, the Th1/Th2 ratio was decreased (*p* < 0.001; Figure 1J), while the Th17/Treg ratio was increased (*p* < 0.01; Figure 1K) in the Model group vs. the Sham group. These data revealed that MALT1 was higher and Th1/Th2 and Th17/Treg ratios were dysregulated in the septic mouse model.

### 3.2. Mucosa-associated lymphoid tissue lymphoma translocation protein 1 aggravated organ injury in septic mouse model

**Figure 2:**
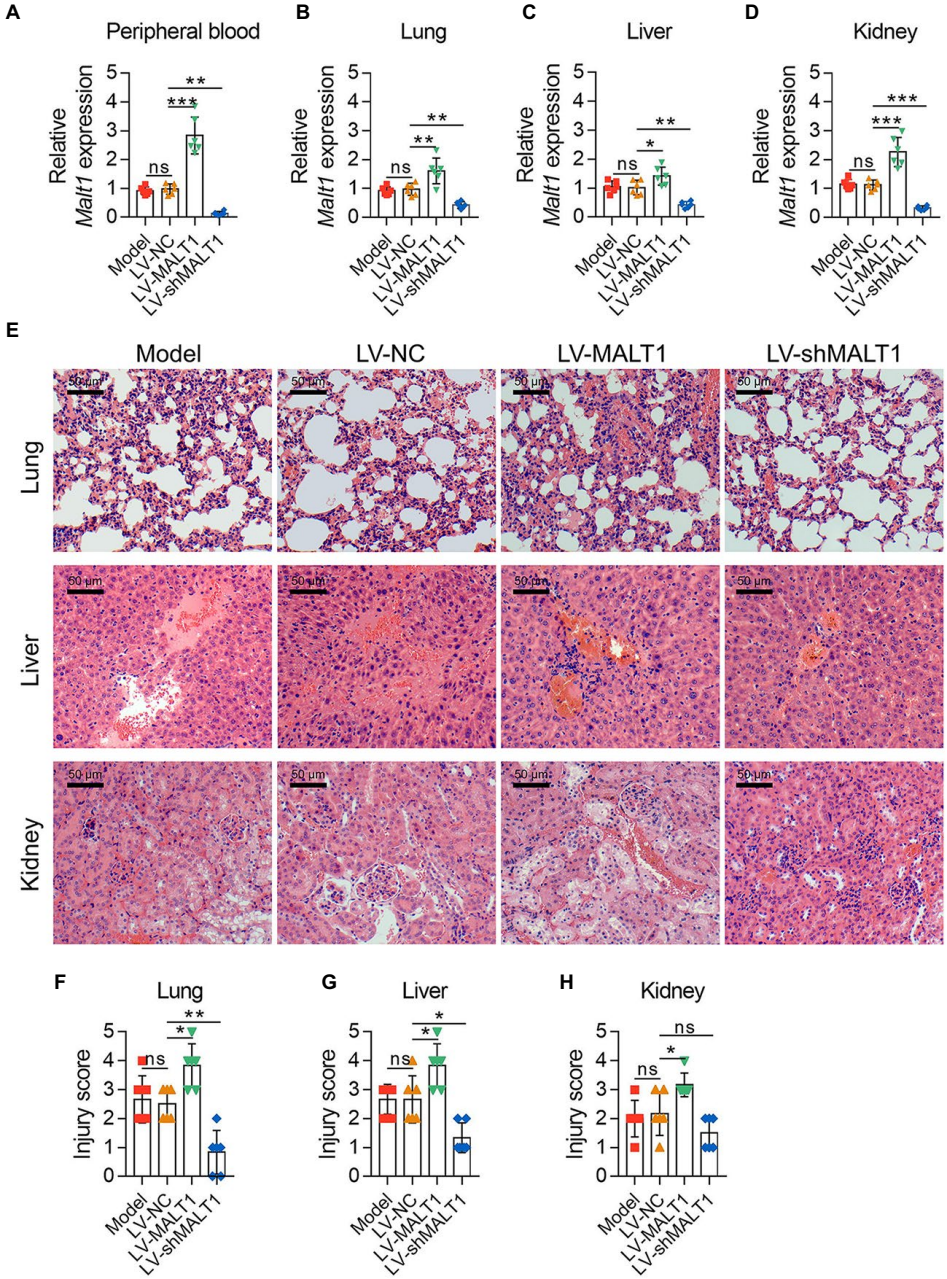
Effect of MALT1 on tissue injury in LPS-induced septic mouse model. Comparison of *Malt1* expression in peripheral blood **(A)**, lung **(B)**, liver **(C)**, and kidney **(D)** among the Model, LV-NC, LV-MALT1, and LV-shMALT1 groups. HE analysis of lung, liver, and kidney tissues among the Model, LV-NC, LV-MALT1, and LV-shMALT1 groups **(E)**. Comparison of injury scores of the lung **(F)**, liver **(G)**, and kidney **(H)** among the Model, LV-NC, LV-MALT1, and LV-shMALT1 groups. n = 6 in each group. One-way ANOVA plus Tukey’s *post hoc* test was applied. Ns, not significant; *p < 0.05; **p < 0.01; ***p < 0.001.

*Malt1* levels in the peripheral blood (both *p* < 0.01; Figure 2A), lung (both *p* < 0.01; Figure 2B), liver (both *p* < 0.05; Figure 2C), and kidney (both *p* < 0.001; Figure 2D) were all increased in the LV-MALT1 group but decreased in the LV-shMALT1 group vs. LV-NC group. The protein level of MALT1 also showed similar trends (all *p* < 0.05; Supplementary Figures S4A–H). Next, HE analysis of lung, liver, and kidney tissues (Figure 2E) revealed that lung, liver, and kidney injuries were higher in the LV-MALT1 group (all *p* < 0.05), while lung and liver injuries were lower in the LV-shMALT1 group (both *p* < 0.05) vs. LV-NC group, but kidney injuries did not vary (*p* > 0.05) between groups (Figures 2F–H).

**Figure 3:**
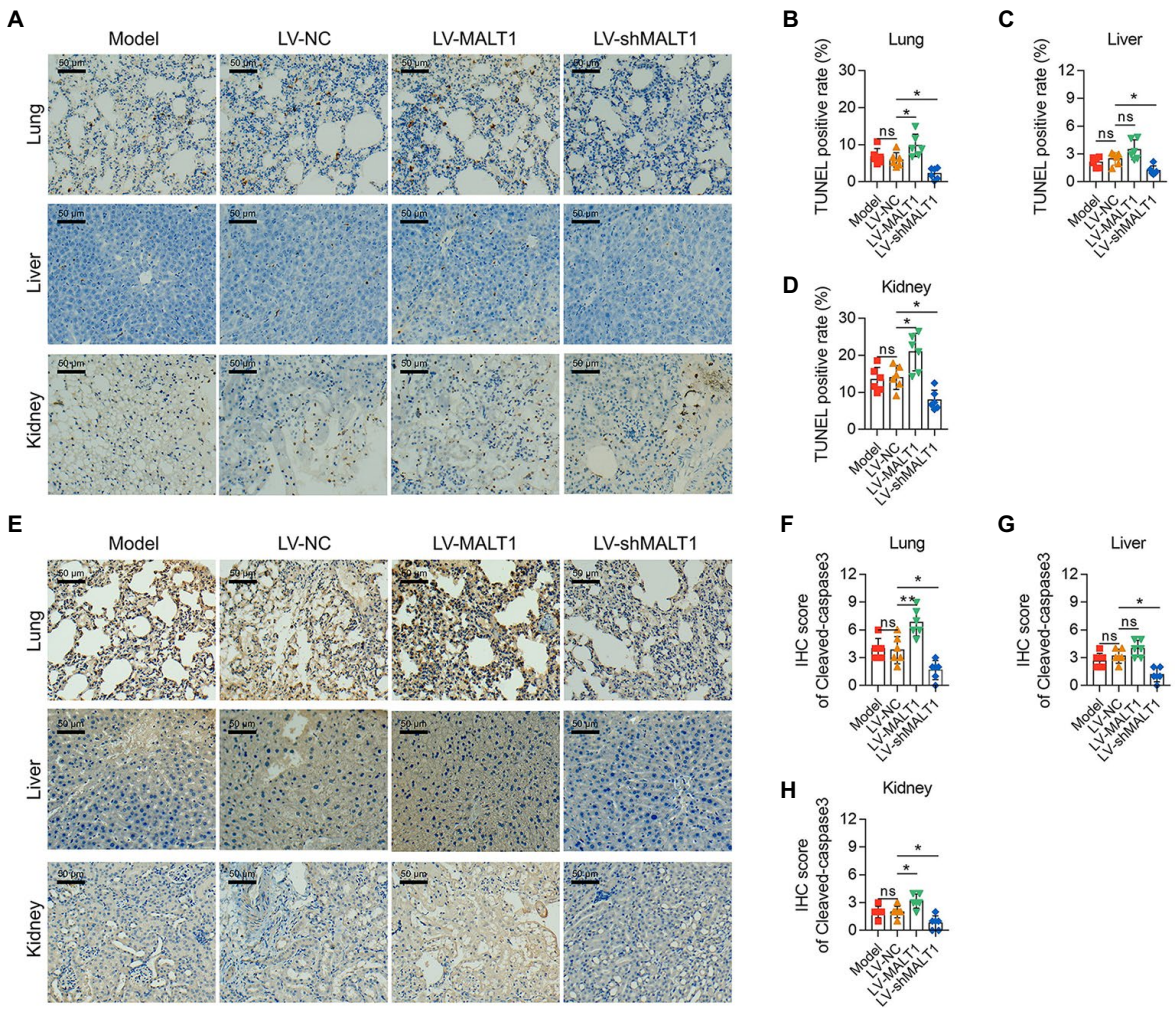
Effect of MALT1 on cell apoptosis in multiple organs of LPS-induced septic mouse model. TUNEL assay of lung, liver, and kidney tissues among the Model, LV-NC, LV-MALT1, and LV-shMALT1 groups **(A)**. Comparison of the TUNEL-positive rate of the lung **(B)**, liver **(C)**, and kidney **(D)** among the Model, LV-NC, LV-MALT1, and LV-shMALT1 groups. IHC analysis of cleaved-caspase3 in lung, liver, and kidney tissues among the Model, LV-NC, LV-MALT1, and LV-shMALT1 groups **(E)**. Comparison of cleaved-caspase3 IHC scores of the lung **(F)**, liver **(G)**, and kidney **(H)** among the Model, LV-NC, LV-MALT1, and LV-shMALT1 groups. *n* = 6 in each group. One-way ANOVA plus Tukey’s *post hoc* test was applied. Ns, not significant; *p < 0.05; **p < 0.01.

In addition, the apoptosis rate of the lung, liver, and kidney was analyzed with a TUNEL assay and IHC staining of cleaved-caspase3. By TUNEL assay (Figure 3A), it was revealed that the LV-MALT1 group showed a higher level of apoptosis in the lung (*p* < 0.05) and kidney (*p* < 0.05) but not the liver (*p* > 0.05), while the LV-shMALT1 group presented a lower level of apoptosis in the lung, liver, and kidney (all *p* < 0.05) vs. the LV-NC group (Figures 3B-D). Meanwhile, IHC staining of cleaved-caspase3 (Figure 3E) showed similar trends: the LV-MALT1 group had a higher IHC score of cleaved-caspase3 in the lung (*p* < 0.01) and kidney (*p* < 0.05) but not in the liver (*p* > 0.05), whereas the LV-shMALT1 group had a lower IHC score of cleaved-caspase3 in the lung, liver, and kidney (all *p* < 0.05) vs. LV-NC group (Figures 3F-H). These data indicated that MALT1 overexpression aggravated lung, liver, and kidney injuries in septic mouse model, while MALT1 knockdown had the reverse effects.

### 3.3. Mucosa-associated lymphoid tissue lymphoma translocation protein 1 enhanced macrophage infiltration in septic mouse model

**Figure 4:**
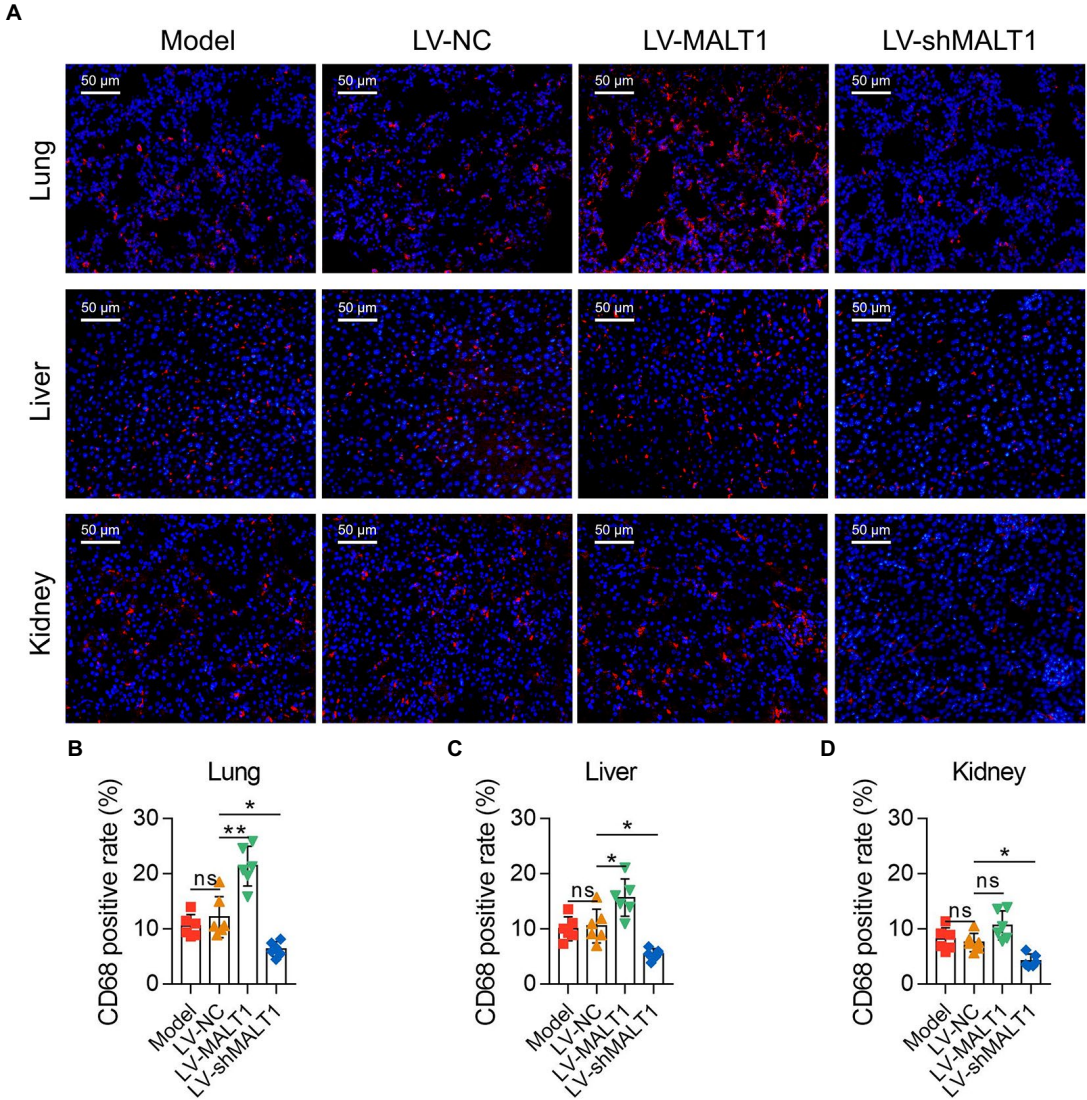
Effect of MALT1 on macrophage infiltration in LPS-induced septic mouse model. IF staining of macrophages in lung, liver, and kidney tissues among the Model, LV-NC, LV-MALT1, and LV-shMALT1 groups **(A)**. Comparison of the CD68-positive rate in the lung **(B)**, liver **(C)**, and kidney **(D)** among the Model, LV-NC, LV-MALT1, and LV-shMALT1 groups. n = 6 in each group. One-way ANOVA plus Tukey’s *post hoc* test was applied. Ns, not significant; *p < 0.05; **p < 0.01.

IF staining of CD68 was applied to analyze macrophage infiltration in the tissues (Figure 4A). The data showed that the LV-MALT1 group had a higher level of macrophage infiltration in the lung (*p* < 0.01) and liver (*p* < 0.05) but not in the kidney (*p* > 0.05), whereas the LV-shMALT1 group had a lower level of macrophage infiltration in the lung, liver, and kidney (all *p* < 0.05) vs. LV-NC group (Figures 4B-D). These data suggested that MALT1 overexpression promoted macrophage infiltration in the lung, liver, and kidney of the septic mouse model, but MALT1 knockdown had the opposite effects.

### 3.4. Mucosa-associated lymphoid tissue lymphoma translocation protein 1 elevated inflammation, oxidative stress, and liver and kidney dysfunction in septic mouse model

**Figure 5:**
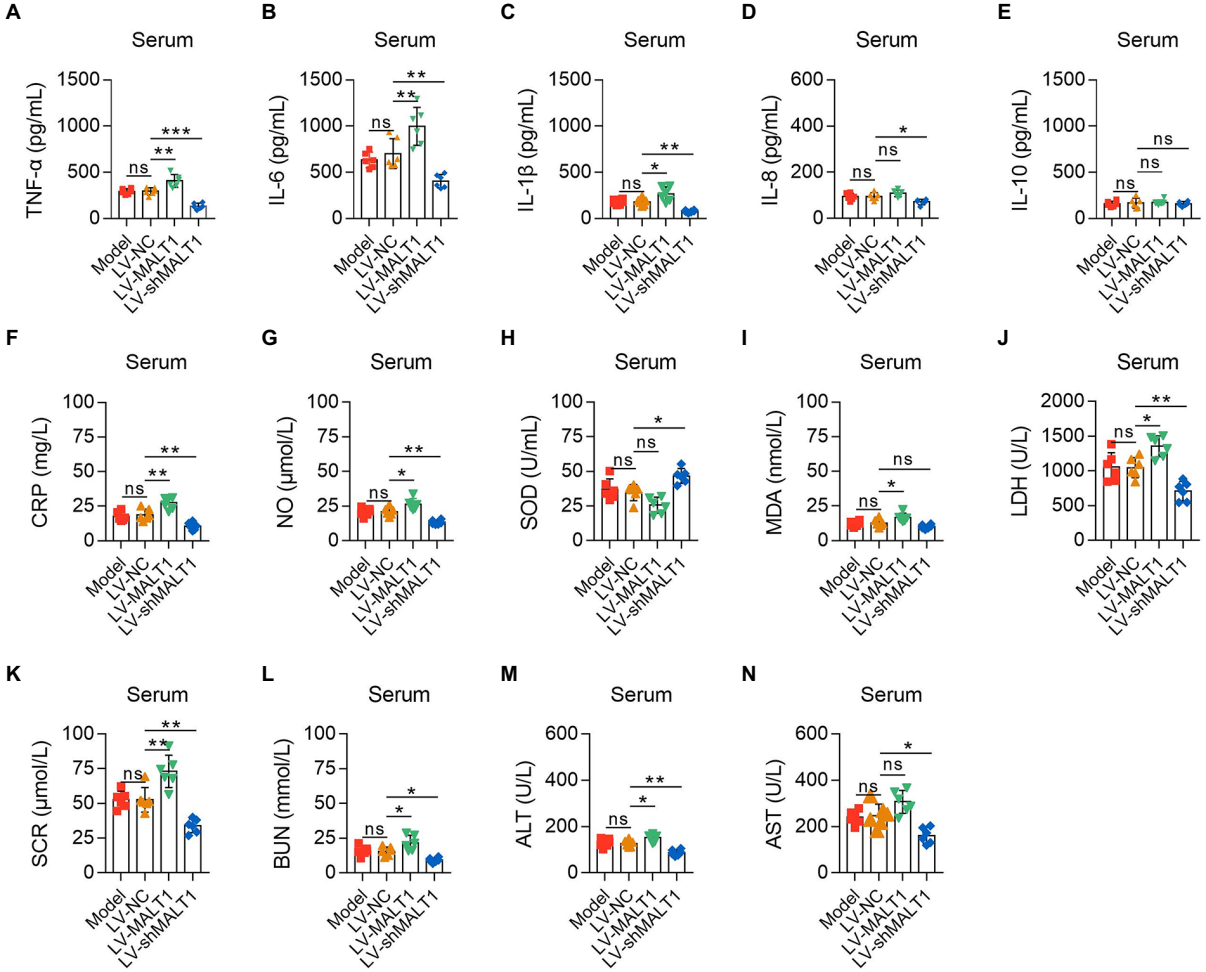
Effect of MALT1 on inflammation, oxidative stress, and liver and kidney function indexes in LPS-induced septic mouse model. Comparison of TNF-α **(A)**, IL-6 **(B)**, IL-1β **(C)**, IL-8 **(D)**, IL-10 **(E)**, CRP **(F)**, NO **(G)**, SOD **(H)**, MDA **(I)**, LDH **(J)**, SCR **(K)**, BUN **(L)**, ALT **(M)**, and AST **(N)** in the serum among the Model, LV-NC, LV-MALT1, and LV-shMALT1 groups. *n* = 6 in each group. One-way ANOVA plus Tukey’s *post hoc* test was applied. Ns, not significant; *p < 0.05; **p < 0.01; ***p < 0.001.

ELISA and automatic animal biochemistry analyzer were adopted to analyze the inflammation, oxidative stress, and liver and kidney dysfunction indexes in the serum. The data showed that the LV-MALT1 group had higher levels of TNF-α (*p* < 0.01), IL-6 (*p* < 0.01), IL-1β (*p* < 0.05), and CRP (*p* < 0.01) but unchanged levels of IL-8 and IL-10 (both *p* > 0.05), while the LV-shMALT1 group presented lower levels of TNF-α (*p* < 0.001), IL-6 (*p* < 0.01), IL-1β (*p* < 0.01), IL-8 (*p* < 0.05), and CRP (*p* < 0.01) but similar levels of IL-10 (*p* > 0.05) vs. LV-NC group (Figures 5A-F). In terms of oxidative stress markers, the levels of NO (*p* < 0.05) and MDA (*p* < 0.05) were increased, but SOD (*p* > 0.05) was not changed in the LV-MALT1 group vs. the LV-NC group, whereas NO (*p* < 0.01) was reduced, SOD (*p* < 0.05) was elevated, but MDA (*p* > 0.05) showed no difference in the LV-shMALT1 group vs. the LV-NC group (Figures 5G-I). Regarding liver and kidney function indexes, LDH (*p* < 0.05), SCR (*p* < 0.01), BUN (*p* < 0.05), and ALT (*p* < 0.05) were elevated, while AST (*p* > 0.05) was not different in the LV-MALT1 group vs. LV-NC group, while LDH (*p* < 0.01), SCR (*p* < 0.01), BUN (*p* < 0.05), ALT (*p* < 0.01), and AST (*p* < 0.05) were all decreased in the LV-shMALT1 group vs. LV-NC group (Figures 5J-N). These data indicated that MALT1 overexpression amplified inflammation, oxidative stress, and liver and kidney injury in septic mouse model, whereas MALT1 knockdown showed the opposite effects.

### 3.5. Mucosa-associated lymphoid tissue lymphoma translocation protein 1 increased Th1/Th2 and Th17/Treg imbalance in septic mouse model

**Figure 6:**
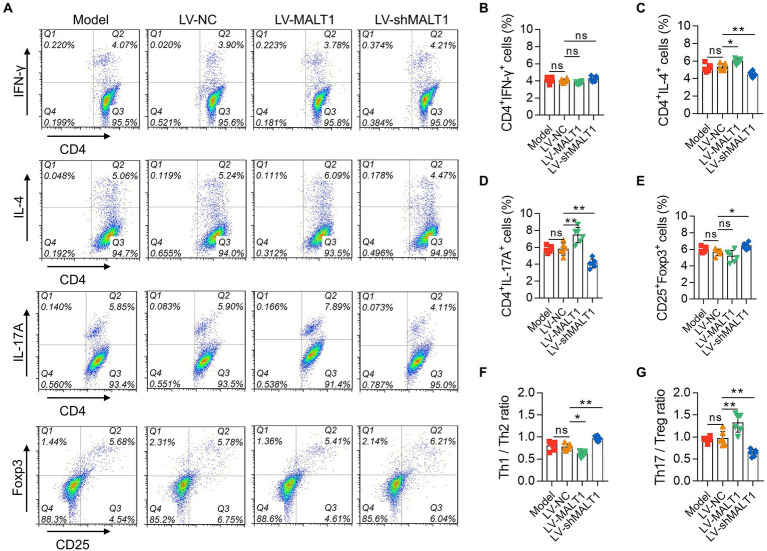
Effect of MALT1 on CD4^+^ T-cell polarization in LPS-induced septic mouse model. Detection of CD4^+^ T cells by flow cytometry **(A)**. Comparison of Th1 **(B)**, Th2 **(C)**, Th17 **(D)**, and Treg **(E)** cells, Th1/Th2 ratio **(F)**, and Th17/Treg ratio **(G)** among the Model, LV-NC, LV-MALT1, and LV-shMALT1 groups. *n* = 6 in each group. One-way ANOVA plus Tukey’s *post hoc* test was applied. Ns, not significant; *p < 0.05; **p < 0.01.

The levels of PBMC Th1, Th2, Th17, and Treg cells were analyzed with flow cytometry (Figure 6A). Compared with the LV-NC group, Th1 cells were not changed in the LV-MALT1 or LV-shMALT1 group (both *p* > 0.05; Figure 6B); Th2 cells were elevated in the LV-MALT1 group (*p* < 0.05) but reduced in the LV-shMALT1 group (*p* < 0.01; Figure 6C); Th17 cells were increased in the LV-MALT1 group (*p* < 0.01) but decreased in the LV-shMALT1 group (*p* < 0.01; Figure 6D). Moreover, Treg cells were not changed in the LV-MALT1 group (*p* > 0.05) but were elevated in the LV-shMALT1 group (*p* < 0.05) vs. the LV-NC group (Figure 6E). Finally, the Th1/Th2 ratio was decreased in the LV-MALT1 group (*p* < 0.05) but elevated in the LV-shMALT1 group (*p* < 0.01; Figure 6F), while the Th17/Treg ratio was increased in the LV-MALT1 group (*p* < 0.01) but decreased in the LV-shMALT1 group (*p* < 0.01) vs. LV-NC group (Figure 6G). These data revealed that MALT1 overexpression intensified Th1/Th2 and Th17/Treg imbalance in septic mouse model, but MALT1 knockdown exerted the converse effects.

### 3.6. Mucosa-associated lymphoid tissue lymphoma translocation protein 1 activated the NF-κB pathway in septic mouse model

**Figure 7:**
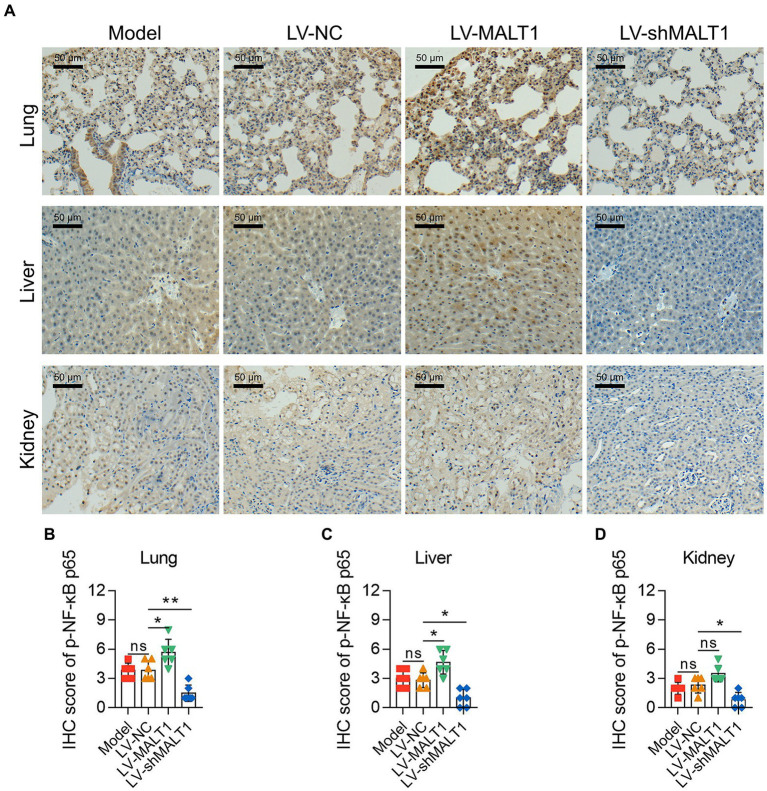
Effect of MALT1 on the NF-κB pathway in LPS-induced septic mouse model. IHC analysis of p-NF-κB p65 in lung, liver, and kidney tissues among the Model, LV-NC, LV-MALT1, and LV-shMALT1 groups **(A)**. Comparison of p-NF-κB p65 IHC scores in the lung **(B)**, liver **(C)**, and kidney **(D)** among the Model, LV-NC, LV-MALT1, and LV-shMALT1 groups. *n* = 6 in each group. One-way ANOVA plus Tukey’s *post hoc* test was applied. Ns, not significant; *p < 0.05; **p < 0.01.

The level of p-NF-κB p65 in the lung, liver, and kidney of septic mice was studied by IHC staining (Figure 7A). The LV-MALT1 group had a higher IHC score of p-NF-κB p65 in the lung and liver (both *p* < 0.05) but not in the kidney (*p* > 0.05); meanwhile, the LV-shMALT1 group presented a lower IHC score of p-NF-κB p65 in the lung (*p* < 0.01), liver (*p* < 0.05), and kidney (*p* < 0.05) vs. LV-NC group (Figures 7B-D). These data showed that MALT1 overexpression activated the NF-κB pathway in septic mouse model, but MALT1 knockdown inhibited this pathway.

### 3.7. Mucosa-associated lymphoid tissue lymphoma translocation protein 1 activated M1 polarization and the NF-κB pathway in lipopolysaccharide-treated bone marrow-derived macrophages

**Figure 8:**
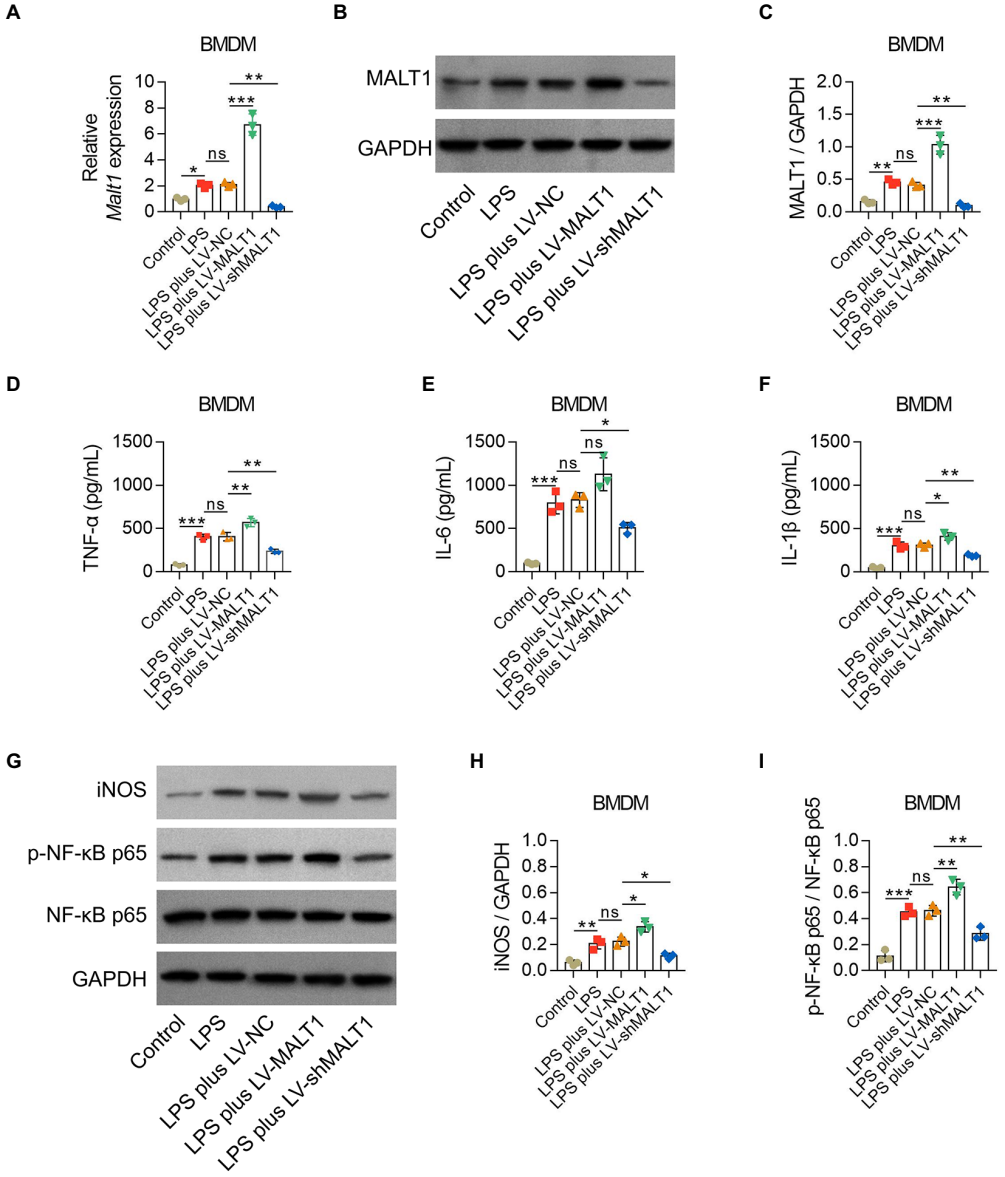
Effect of MALT1 on M1 polarization of LPS-treated macrophages. Comparison of MALT1 mRNA levels **(A)** and protein levels (**B**,**C**) among Control, LPS, LPS plus LV-NC, LPS plus LV-MALT1, and LPS plus LV-shMALT1 groups. Comparison of TNF-α (**D**), IL-6 **(E)**, and IL-1β **(F)** among Control, LPS, LPS plus LV-NC, LPS plus LV-MALT1, and LPS plus LV-shMALT1 groups. Western blot detection of iNOS and p-NF-κB p65 **(G)** and comparison of the relative protein levels of iNOS **(H)** and p-NF-κB p65 **(I)** among Control, LPS, LPS plus LV-NC, LPS plus LV-MALT1, and LPS plus LV-shMALT1 groups. n = 3 in each group. One-way ANOVA plus Tukey’s *post hoc* test was applied. Ns, not significant; *p < 0.05; **p < 0.01; ***p < 0.001.

After LPS stimulation, BMDMs showed higher MALT1 mRNA and protein levels (both *p* < 0.05), inflammatory cytokines including TNF-α, IL-6, and IL-1β (all *p* < 0.001), and iNOS and p-NF-κB p65 levels (both *p* < 0.01; Figures 8A-I).

Subsequently, BMDMs were treated with LPS combined with LV-NC, LV-MALT1, or LV-shMALT1. MALAT1 mRNA and protein levels were elevated in LV-MALT1-treated cells (both *p* < 0.001) but declined in LV-shMALT1-treated cells (both *p* < 0.01) vs. LV-NC-treated cells (Figures 8A-C), which suggested the success of lentivirus transfection. Meanwhile, LV-MALT1-treated cells showed higher levels of TNF-α and IL-1β (both *p* < 0.05) but not IL-6 (*p* > 0.05), whereas LV-shMALT1-treated cells had lower levels of TNF-α, IL-6, and IL-1β (all *p* < 0.05) vs. LV-NC-treated cells (Figures 8D-F). Moreover, the protein levels of iNOS and p-NF-κB p65 were both increased in LV-MALT1-treated cells (both *p* < 0.05) but reduced in LV-shMALT1-treated cells (both *p* < 0.05) vs. LV-NC-treated cells (Figures 8G-I). These data suggested that MALT1 overexpression enhanced M1 polarization of macrophages in sepsis, while MALT1 knockdown had the opposite effect.

### 3.8. Mucosa-associated lymphoid tissue lymphoma translocation protein 1 promoted Th2 and Th17 polarization and activated the NF-κB pathway in lipopolysaccharide-treated naïve CD4^+^ T cells

**Figure 9:**
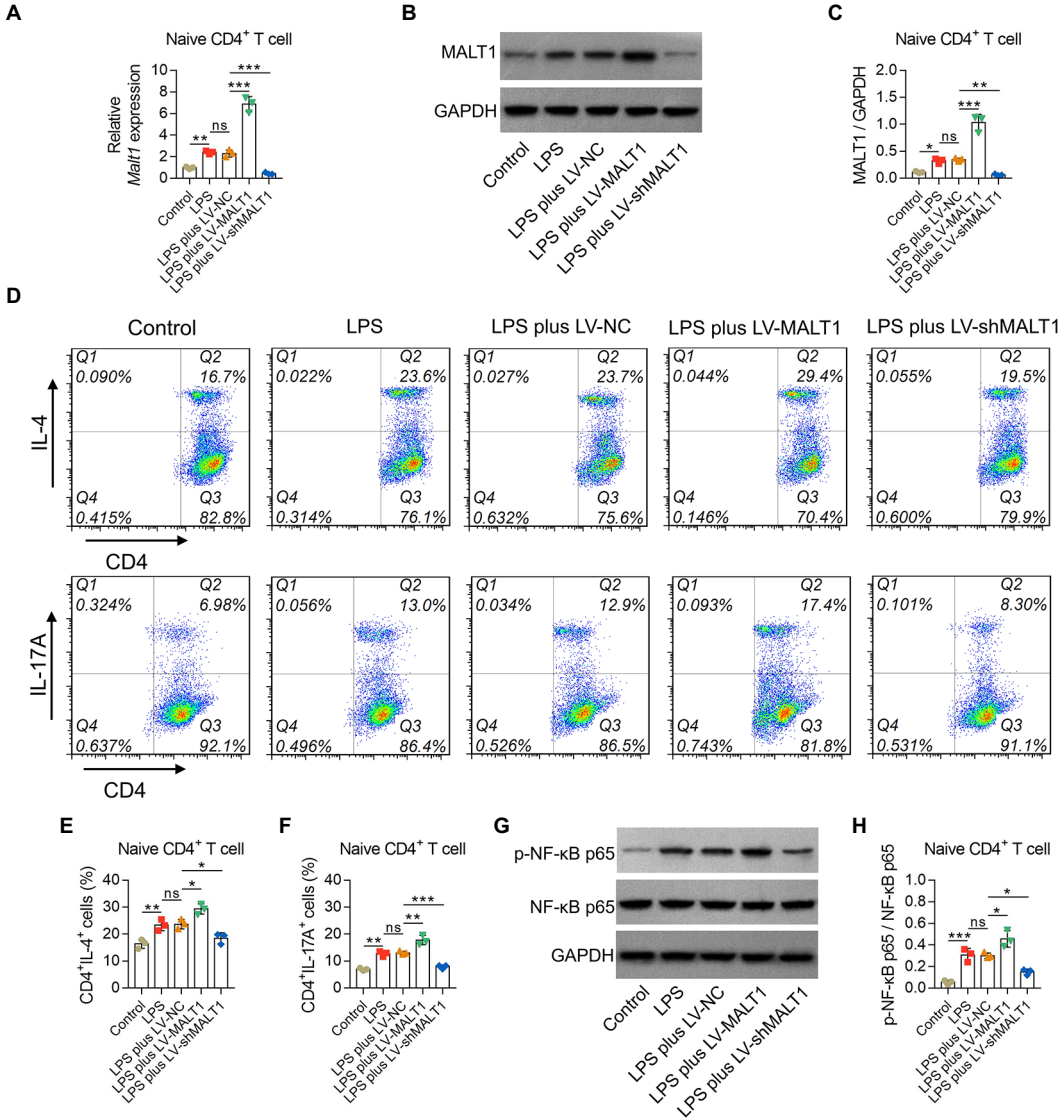
Effect of MALT1 on the polarization of LPS-treated naïve CD4^+^ T cells. Comparison of MALT1 mRNA levels **(A)** and protein levels (**B**,**C**) among Control, LPS, LPS plus LV-NC, LPS plus LV-MALT1, and LPS plus LV-shMALT1 groups. Flow cytometry analysis of Th2 and Th17 cells among Control, LPS, LPS plus LV-NC, LPS plus LV-MALT1, and LPS plus LV-shMALT1 groups (**D**). Comparison of Th2 **(E)** and Th17 **(F)** cell proportions among Control, LPS, LPS plus LV-NC, LPS plus LV-MALT1, and LPS plus LV-shMALT1 groups. Western blot detection of p-NF-κB p65 **(G)** and comparison of the relative protein level of p-NF-κB p65 **(H)** among Control, LPS, LPS plus LV-NC, LPS plus LV-MALT1, and LPS plus LV-shMALT1 groups. n = 3 in each group. One-way ANOVA plus Tukey’s *post hoc* test was applied. Ns, not significant; *p < 0.05; **p < 0.01; ***p < 0.001.

LPS treatment induced higher MALT1 mRNA and protein levels (both *p* < 0.05), elevated the proportions of Th2 and Th17 cells (both *p* < 0.01), and promoted p-NF-κB p65 levels (*p* < 0.001) in naïve CD4^+^ T cells (Figures 9A-H).

Then, naïve CD4^+^ T cells were treated with LPS and LV-NC, LV-MALT1, or LV-shMALT1. The data showed that MALT1 mRNA and protein levels were elevated in LV-MALT1-treated cells (both *p* < 0.001) but declined in LV-shMALT1-treated cells (both *p* < 0.01) vs. LV-NC-treated cells (Figures 9A-C). After analysis by flow cytometry (Figure 9D), it was found that Th2 and Th17 proportions were elevated in LV-MALT1-treated cells (both *p* < 0.05) but declined in LV-shMALT1-treated cells (both *p* < 0.05) vs. LV-NC-treated cells (Figures 9E,F). Moreover, p-NF-κB p65 was increased in LV-MALT1-treated cells (*p* < 0.05) but decreased in LV-shMALT1-treated cells (*p* < 0.05) vs. LV-NC-treated cells (Figures 9G,H). These data illustrated that MALT1 overexpression elevated Th2 and Th17 polarization in sepsis, but MALT1 knockdown showed the opposite effects.

## 4. Discussion

Sepsis is a critical health issue that needs to be solved, and the management modalities of sepsis are still unsatisfying (Alhazzani et al., 2020; Rudd et al., 2020). The current study revealed that MALT1 exaggerated lung, liver, and kidney injuries in a septic mouse model. Meanwhile, MALT1 promoted the polarization of M1 macrophages, Th2 cells, and Th17 cells in sepsis. Moreover, MALT1 exerted these effects by activating the NF-κB pathway in sepsis. These findings highlight the involvement of MALT1 in the pathogenesis and progression of sepsis, which might serve as a potential target for the management of sepsis.

Multiple organ dysfunction is one of the hallmarks of sepsis (Cecconi et al., 2018). In patients with sepsis, injuries of the lung, liver, kidney, anticoagulant system, heart, nervous system, etc., are likely to occur, which are closely associated with the mortality risk of sepsis (Zhou and Liao, 2021; Lu et al., 2022). Previously, some studies have shown that MALT1 is a potential regulator of organ dysfunction. For instance, it has been reported that inhibition of MALT1 reduces tissue injury in pulmonary fibrosis model mice (Fusco et al., 2020). Another study revealed that blockage of MALT1 alleviates the endoplasmic reticulum of glia in spinal cord ischemia/reperfusion injury and downregulates M1 polarization of microglia in the spinal cord (Zhang et al., 2021). Moreover, suppression of the MALT1-dependent NF-κB pathway reduces inflammation levels in angiotensin II-induced chronic liver injury model mice (McAllister-Lucas et al., 2007). In terms of the regulation of MALT1 in sepsis, although a recent study revealed its positive correlation with multiple organ injury in patients with sepsis (Wang et al., 2022), whether MALT1 exerts a regulatory effect on sepsis-induced organ injury remains unclear. In the current study, septic mouse model was constructed by intraperitoneal injection of LPS; then, MALT1 overexpression exaggerated the injuries and apoptosis of the lung, liver, and kidney, while MALT1 knockdown exerted the opposite effects. Possible explanations might be that (1) MALT1 could activate the NF-κB pathway, which induced apoptosis in the epithelial cells of the lung, liver, and kidney and finally caused organ injury (Ekambaram et al., 2018; Sangaran et al., 2021). (2) MALT1 might activate T-cell receptor (TCR) signaling, which also promotes organ injury (Govender et al., 2020; Mustafa et al., 2021). (3) MALT1 could elevate inflammation and oxidative stress, which might exaggerate organ injury as well (Gong et al., 2022; He et al., 2022). However, MALT1 mRNA expression in the liver was not changed in the septic mouse model compared with Sham group, suggesting that the liver might not be the target of septic pathogenesis and progression by MALT1. The liver injury score was altered by MALT1 modification, indicating that liver injury might come from a second attack of sepsis. Therefore, further conditioned MALT1 modification should be conducted to illustrate the target of septic pathogenesis and progression by MALT1.

The current study also investigated the effect of MALT1 on inflammation and oxidative stress in septic model mice. Inflammation is a key parameter of sepsis, which is mediated by the dysregulation of immune cells and release of proinflammatory cytokines (Cecconi et al., 2018). Oxidative stress contributes to inflammation and organ injury during sepsis (Kumar et al., 2022). Various studies have disclosed that inhibition of oxidative stress ameliorates sepsis and/or sepsis-induced multiple organ dysfunctions, including lung, liver, kidney, heart, etc (Gong et al., 2022; He et al., 2022; Li X. et al., 2022; Li L. et al., 2022). The data of our study revealed that MALT1 overexpression increased the levels of serum proinflammatory cytokines, including TNF-α, IL-6, and IL-1β, while MALT1 knockdown decreased these proinflammatory cytokines. In addition, the markers of oxidative stress, including NO and MDA, were elevated, but the antioxidant enzyme SOD was reduced by MALT1 overexpression; however, MALT1 knockdown showed the opposite effect. These findings could be explained by the following: MALT1 could activate the NF-κB pathway (Ekambaram et al., 2018); meanwhile, the NF-κB pathway critically increases the levels of inflammation and oxidative stress (Capece et al., 2022; Kumar et al., 2022). MALT1 might promote the activation of TCR signaling, which is a vital inducer of both inflammation and oxidative stress (Mustafa et al., 2021). MALT1 could increase the polarization of M1 macrophages and Th17 cells, which release proinflammatory cytokines such as TNF-α, IL-1, and IL-17, thus resulting in a higher level of inflammation (Wang et al., 2019; Meitei and Lal, 2022). However, IL-10 showed no difference with MALT1 overexpression or knockdown. A possible explanation might be that IL-10 was mainly released by M2 macrophages and Th2 cells (Rasquinha et al., 2021; Cutolo et al., 2022). From the data of this study, Th2 cell polarization was promoted by MALT1, while M2 macrophages could be inhibited by MALT1, which might result in unchanged IL-10 levels in septic mouse model.

The dysregulation of immune cells, including macrophages and CD4^+^ T cells, is also a vital pathogenesis of sepsis (Cecconi et al., 2018). Macrophages can be polarized into two different phenotypes, M1 and M2 macrophages, among which M1 macrophages mainly secrete cytokines such as TNF-α and IL-1 to induce inflammation, thus protecting against microbiome infection, whereas M2 macrophages exert protection against parasitic infection and wound healing effects (Wang et al., 2019). Naïve CD4^+^ T cells can polarize into Th1, Th2, Th17, Treg, etc., and exert immune regulatory effects (Meitei and Lal, 2022). During sepsis, these immune cells are dysregulated, which induces inflammation and potentially causes organ dysfunction (Martin et al., 2020; Chen et al., 2021). According to previous studies, MALT1 is able to regulate the polarization of macrophages and CD4^+^ T cells (Biswas et al., 2022; Wang et al., 2022; Xu et al., 2022); however, whether similar regulatory effects also exist under sepsis conditions is still unclear. In the current study, MALT1 overexpression promoted macrophage infiltration in the lung, liver, and kidney, as well as the Th17/Treg ratio, while decreasing the Th1/Th2 ratio; however, MALT1 knockdown showed the opposite effect in sepsis model mice. In addition, MALT1 overexpression increased *ex vivo* polarization of M1 macrophages, Th2 cells, and Th17 cells under LPS treatment, while MALT1 knockdown showed the opposite effects. A possible explanation for these data might be that (1) MALT1 could activate TCR signaling and its related NF-κB pathway to facilitate the polarization of M1 macrophages, Th2 cells, and Th17 cells (Yamane and Paul, 2013; Wang et al., 2022; Xu et al., 2022). (2) MALT1 might also promote the production of IL-6 through NF-κB activation, which further triggers the signaling transducer and activator of transcription 3 (STAT3) pathway to regulate macrophage and CD4^+^ T-cell polarization (Wang et al., 2018).

The NF-κB pathway is a critical signaling pathway that modulates the pathogenesis and progression of sepsis. As mentioned above, the NF-κB pathway is able to regulate cell apoptosis, inflammation, oxidative stress, and polarization of macrophages and CD4^+^ T cells (Ekambaram et al., 2018; Kumar et al., 2022; Wang et al., 2022; Xu et al., 2022), which are critical mechanisms of sepsis (Cecconi et al., 2018). Meanwhile, it has also been reported that the NF-κB pathway is activated in an LPS-induced septic mouse model and in septic patients (Cao et al., 2020; Ke and Cai, 2021). According to previous studies, MALT1 is a vital regulator of the NF-κB pathway (Schaefer, 2020). MALT1, together with the caspase recruitment domain-containing (CARD) family and B-cell lymphoma 10 (BCL10), forms the CARD-BCL10-MALT1 (CBM) complex that mediates the activation of the NF-κB transcription factor in various pathological conditions, including malignancies, allergic response, and infection (McAuley et al., 2019; Alfano et al., 2020; Chang et al., 2022). However, the interaction between MALT1 and the NF-κB pathway in sepsis has yet to be elucidated. In the current study, it was found that the NF-κB pathway in lung, liver, and kidney tissues of septic model mice was activated by MALT1 overexpression but inhibited by MALT1 knockdown. Moreover, similar trends were also observed in *ex vivo* LPS-treated macrophages and naïve CD4^+^ T cells. Together with the abovementioned findings, it is assumed that the NF-κB pathway might be implicated in the regulation of MALT1 in sepsis. However, this should be further verified.

The current study had several limitations. First, the septic mouse model was constructed based on LPS derived from *E. coli*, while patients with sepsis might be infected with other microbes. Therefore, the findings of the current study should be further verified. Second, potential confounding factors existed in the current study, such as the order in which animals were processed. Third, the target organ of MALT1 that induces the pathogenesis and progression of sepsis was not explored, which should be further verified with conditioned MALT1 modification in a septic mouse model.

Conclusively, MALT1 exacerbates multiple organ injury, inflammation, M1 macrophage polarization, and Th1/Th2 and Th17/Treg ratio imbalance by activating the NF-κB pathway in sepsis. These findings imply the possibility of targeting MALT1 for the treatment of sepsis.

## Data availability statement

The original contributions presented in the study are included in the article/Supplementary material, further inquiries can be directed to the corresponding author.

## Ethics statement

The animal study was reviewed and approved by Animal Ethics Committee of the Central Hospital of Wuhan.

## Author contributions

FA substantially contributed to the conception and design of the study. YW and ZL were responsible for the acquisition, analysis, and interpretation of the data. MZ and BY contributed to manuscript drafting and critical revisions of the intellectual content. All authors approved the final manuscript to be published.

## Conflict of interest

The authors declare that the research was conducted in the absence of any commercial or financial relationships that could be construed as a potential conflict of interest.

## Publisher’s note

All claims expressed in this article are solely those of the authors and do not necessarily represent those of their affiliated organizations, or those of the publisher, the editors and the reviewers. Any product that may be evaluated in this article, or claim that may be made by its manufacturer, is not guaranteed or endorsed by the publisher.
