# Supplementary material for: Mucosa-associated lymphoid tissue lymphoma translocation protein 1 exaggerates multiple organ injury, inflammation, and immune cell imbalance by activating the NF-κB pathway in sepsis
Source: Front Microbiol. 2023 Mar 7;14:1117285. doi: 10.3389/fmicb.2023.1117285 (PMC10027914; doi:10.3389/fmicb.2023.1117285)
Supplement: Supplementary file 1 [file Data_Sheet_1.PDF]

Supplementary Materials

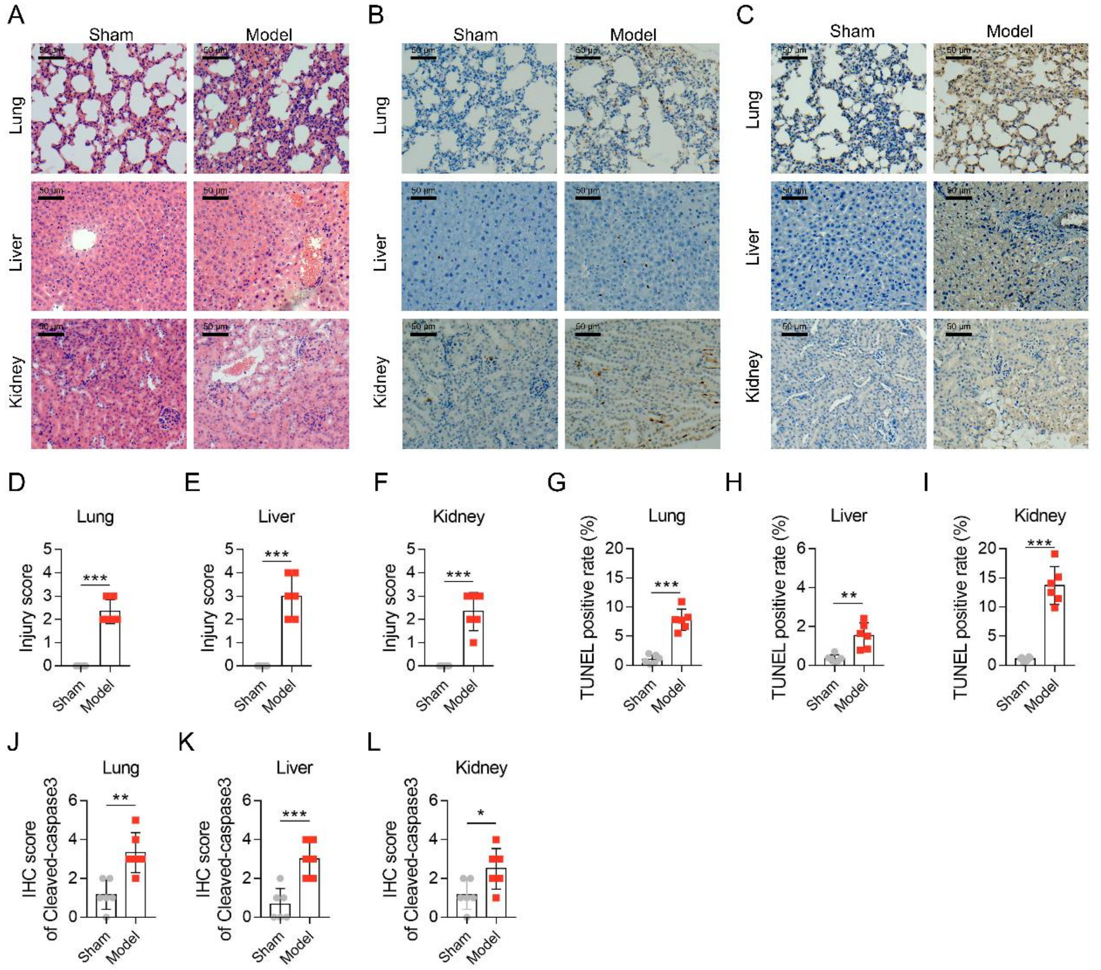

Supplementary Figure 1. Organ injury in LPS-induced septic model mice. HE analysis (**A**), TUNEL assay (**B**), and IHC analysis of cleaved caspase3 (**C**) in lung, liver, and kidney tissues between the Model and Sham groups. Comparison of injury scores of lung (**D**), liver (**E**), and kidney (**F**), TUNEL positive rate of lung (**G**), liver (**H**), and kidney (**I**), and IHC scores of cleaved-caspase3 in lung (**J**), liver (**K**), and kidney (**L**) between Model and Sham groups. n=6 in each group. The Mann–Whitney U test was applied for panels **D**, **E**, and **F**. Student's t test was applied for panels **G**, **H**, **I**, **J**, **K**, and **L**. *, $P<0.05$; **, $P<0.01$; ***, $P<0.001$

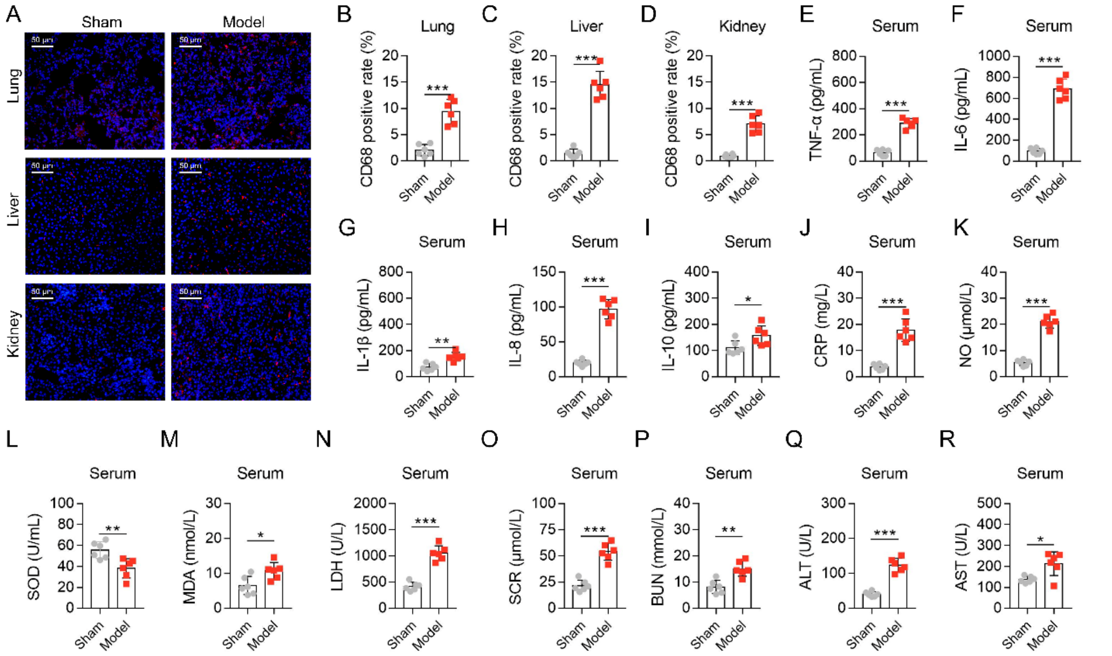

Supplementary Figure 2. Macrophage infiltration, inflammation, oxidative stress, and liver and kidney function indexes in LPS-induced septic mouse model. IF staining of macrophages in lung, liver, and kidney tissues in the Model and Sham groups (**A**). Comparison of CD68 positive rate in lung (**B**), liver (**C**), and kidney (**D**), TNF-α (**E**), IL-6 (**F**), IL-1β (**G**), IL-8 (**H**), IL-10 (**I**), CRP (**J**), NO (**K**), SOD (**L**), MDA (**M**), LDH (**N**), SCR (**O**), BUN (**P**), ALT (**Q**), and AST (**R**) in the serum between Model and Sham groups. n=6 in each group. Student's t test was applied. *, P<0.05; **, P<0.01; ***, P<0.001

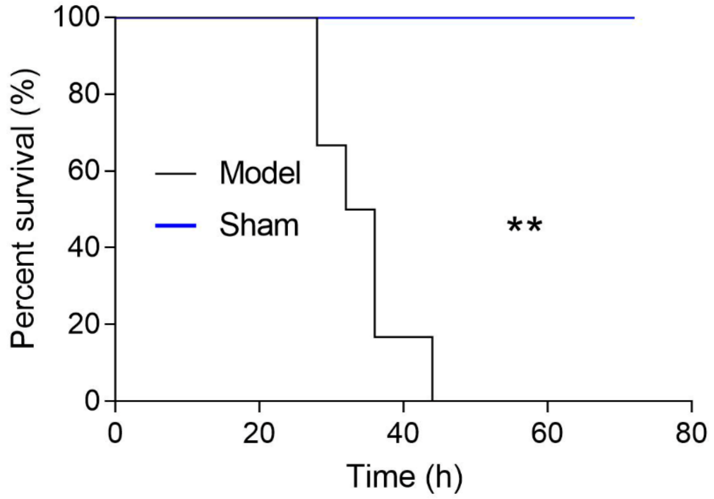

Supplementary Figure 3. Survival of the Model and Sham groups. n=6 in each group. The log-rank test was applied. **, P<0.01

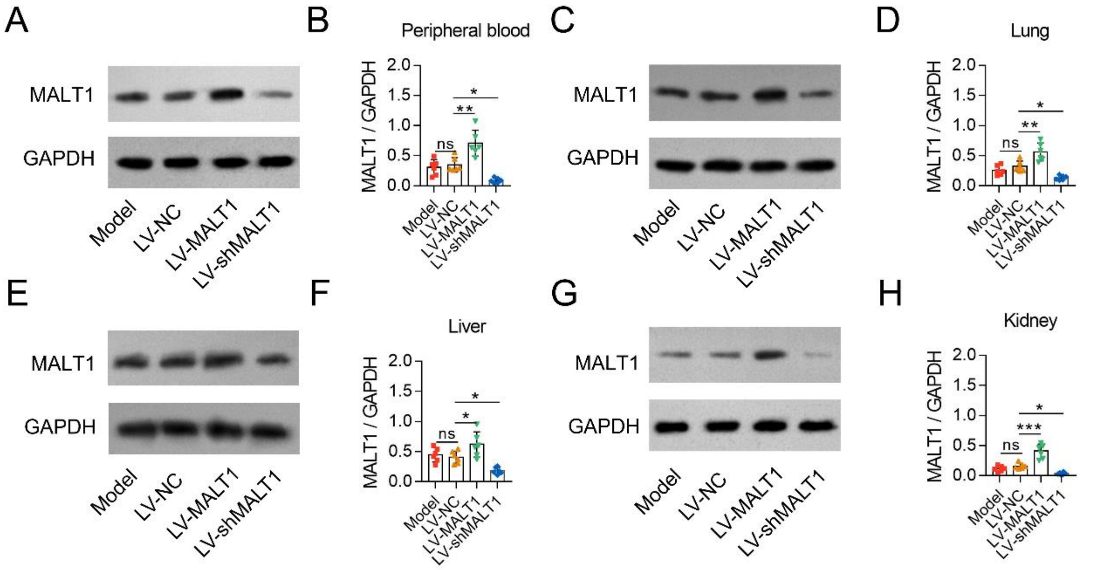

Supplementary Figure 4. MALT1 protein expression in tissues of the septic mouse model after modification of MALT1. Comparison of MALT1 protein expression in peripheral blood (**A, B**), lung (**C, D**), liver (**E, F**), and kidney (**G, H**) among the Model, LV-NC, LV-MALT1, and LV-shMALT1 groups. n=6 in each group. One-way ANOVA plus Tukey's post hoc test was applied. Ns, not significant; *, P<0.05; **, P<0.01; ***, P<0.001
